# A polymethoxyflavone from *Laggera pterodonta* induces apoptosis in imatinib-resistant K562R cells via activation of the intrinsic apoptosis pathway

**DOI:** 10.1186/s12935-014-0137-1

**Published:** 2014-12-05

**Authors:** Changshu Cao, Bailian Liu, Chengwu Zeng, Yuhong Lu, Shaohua Chen, Lijian Yang, Bo Li, Yaolan Li, Yangqiu Li

**Affiliations:** Department of Human Anatomy, Medical School of Jinan University, Guangzhou, 510632 China; Institute of Hematology, Jinan University, Guangzhou, 510632 China; Institute of Traditional Chinese Medicine & Natural Products, College of Pharmacy, Jinan University, Guangzhou, China; Department of Hematology, the First Affiliated Hospital of Jinan University, Guangzhou, 510632 China; Key Laboratory for Regenerative Medicine of Ministry of Education, Jinan University, Guangzhou, 510632 China

**Keywords:** DHTMF, Imatinib-resistant K562 cells, Apoptosis, Cell proliferation

## Abstract

**Background:**

Treatment with imatinib mesylate (IM) (a tyrosine kinase inhibitor) is the first line of standard care for patients newly diagnosed with CML. Despite the success of IM and other tyrosine kinase inhibitors (TKIs), chronic myeloid leukemia (CML) remains largely incurable, and a number of CML patients die due to Abl mutation-related drug resistance and blast crisis. 3, 5-Dihydroxy-6, 7, 3′4′-tetramethoxyflavone (DHTMF) is a polymethoxyflavone isolated from *Laggera pterodonta* which is a herbal medicine used to treat cancer in the Chinese folk. In the previous study, we found DHTMF demonstrated good antiproliferative activities against a number of cancer cell lines and induced the apoptosis of CNE cells *in vitro* in a time- and dose-dependent manner while exhibiting low cytotoxicity in the two normal cell lines Vero and EVC304. The aim of the present study was to evaluate the proliferation inhibition and apoptosis induced by DHTMF alone and in combination with IM in the IM-resistant CML cell line K562R.

**Methods:**

Cell proliferation was assayed with the cell counting kit-8 (CCK8) method. The apoptosis percentage was determined by flow cytometry (FCM). Mitochondrial transmembrane potential was detected using FCM and confocal laser-scanning microscopy. The level of proteins involved in apoptosis was detected by Western blotting.

**Results:**

DHTMF suppressed K562R cell viability in both time- and dose-dependent manners. DHTMF combined with IM enhanced the inhibitory effects and apoptosis in K562R cells as compared with DHTMF alone. DHTMF alone and in combination with IM significantly decreased the mitochondrial membrane potential and increased the levels of cleaved caspase-9, caspase-7, caspase-3, and PARP in K562R cells.

**Conclusions:**

We demonstrated that DHTMF could inhibit IM-resistant K562R cell proliferation and induces apoptosis via the intrinsic mitochondrial apoptotic pathway. These results suggest that DHTMF may be a potential therapeutic drug with lower side effects against IM resistance in CML cells.

## Background

Chronic myeloid leukemia (CML) is a hematopoietic stem cell disorder that occurs due to t (9; 22) (q34; q11) translocations. CML represents approximately 20% of all adult leukemia cases [1]. The aberrant Philadelphia chromosome has been reported to be the main cause of CML development due to fusion with the Bcr-Abl oncogene. The chimeric gene Bcr-Abl encodes a protein with constitutive tyrosine-kinase (TK) activity [2]. CML prognoses have markedly improved after the introduction of Abl tyrosine kinase inhibitors (TKIs). Since approved as frontline CML management in 2001, imatinib mesylate (IM) has been proven to be effective in achieving high remission rates and improving prognosis. However, up to 33% of patients did not achieve an optimal response [3], because residual CML cells were generally present in the bone marrow microenvironment and then refractory to IM [4]. Unfortunately, most CML patients who were treated with IM undergone relapse once the drug was withdrawn, and Abl mutation-related drug resistance and blast crisis resulted in numerous CML patients death [5]. Next-generation TKIs, such as dasatinib and nilotinib, as well as other kinase inhibitors including Janus kinase 2 inhibitor and Bruton's tyrosine kinase (BTK) inhibitor, have been used to overcome IM-resistant cases [6,7]. Despite the increasing success of new TKIs, CML remains largely incurable, and the development of inducible drug resistance is a paramount problem in which patients failed respond to the drugs. How to treat the patients who were resistant to Bcr-Abl tyrosine kinase inhibitors is an important and urgent issue for clinical hematology. Thus, more efforts have been directly focused on developing new drugs to control CML with IM resistance.

Chinese herbal medicines traditionally used to treat cancer are an important source of potential anti-cancer agents [8–11]. *Laggera pterodonta* is an herbal medicine which is used for a long time in Chinese folk for the treatment of various inflammations as well as cancers [12]. Naturally occurring flavonoids have been proved to possess a wide range of biological activities including antitumor activity [13]. Studies have revealed that quite a few flavonoids could reverse drug resistance through different apoptosis pathways [14–16]. In our previous study, a polymethoxyflavone, 3,5-dihydroxy-6,7,3′4′-tetramethoxyflavone (DHTMF), isolated from *Laggera pterodonta* was found to possess good anti-cancer activity [17]. Recently, polymethoxyflavones are gaining increasing attention due to their promising anticancer potential. In this study, we investigated the proliferation inhibition and apoptosis induced by DHTMF alone and in combination with IM in the IM-resistant CML cell line K562R.

## Results

### Effect of DHTMF on cell proliferation

**Figure 1:**
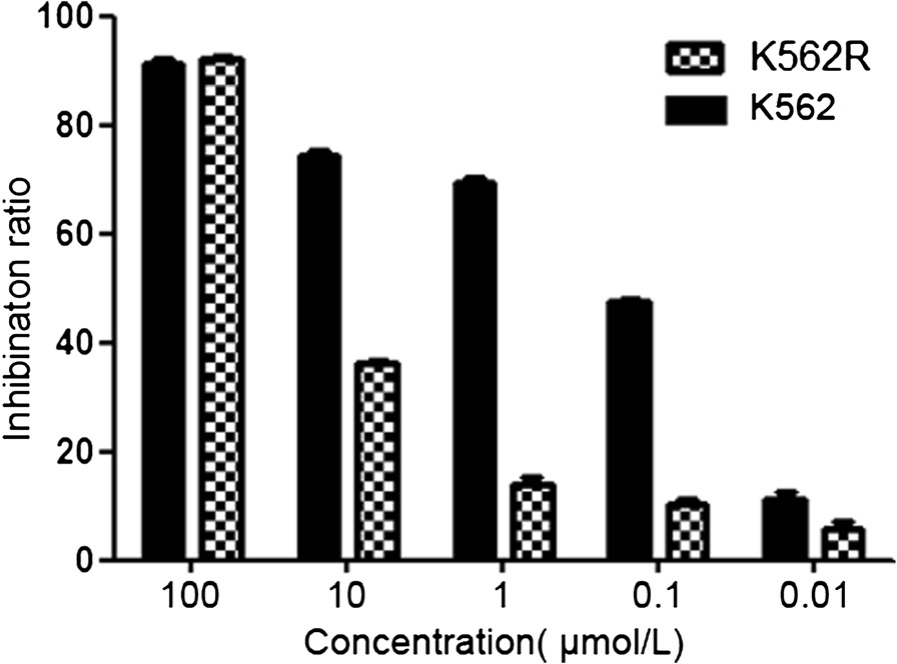
**The inhibitory effect of IM in K562 and K562R cells at 24 h.**

We first verified that the K562R cells we used are IM-resistant CML cells. After K562 and K562R cells were treated with different concentrations of IM for 24 h, their cell viability was determined by the CCK8 assay. The data indicated that IM preferentially inhibits the proliferation of IM-sensitive K562 cells. After the K562 and K562R cells were treated with 1 μmol/L IM for 24 h, the inhibitory ratio for the K562 cells was 69.75, while that for K562R was only 13.99 (Figure 1). We calculated the IC_50_ (50% inhibition concentration) for the K562 cells to be 0.43 μmol/L, and the IC_50_ for the K562R cells to be 6.23 μmol/L, which indicated that K562 cells have a markedly lower IC_50_ compared with K562R cells. The IM resistance fold-change of the K562R cells was 14.49.

**Figure 2:**
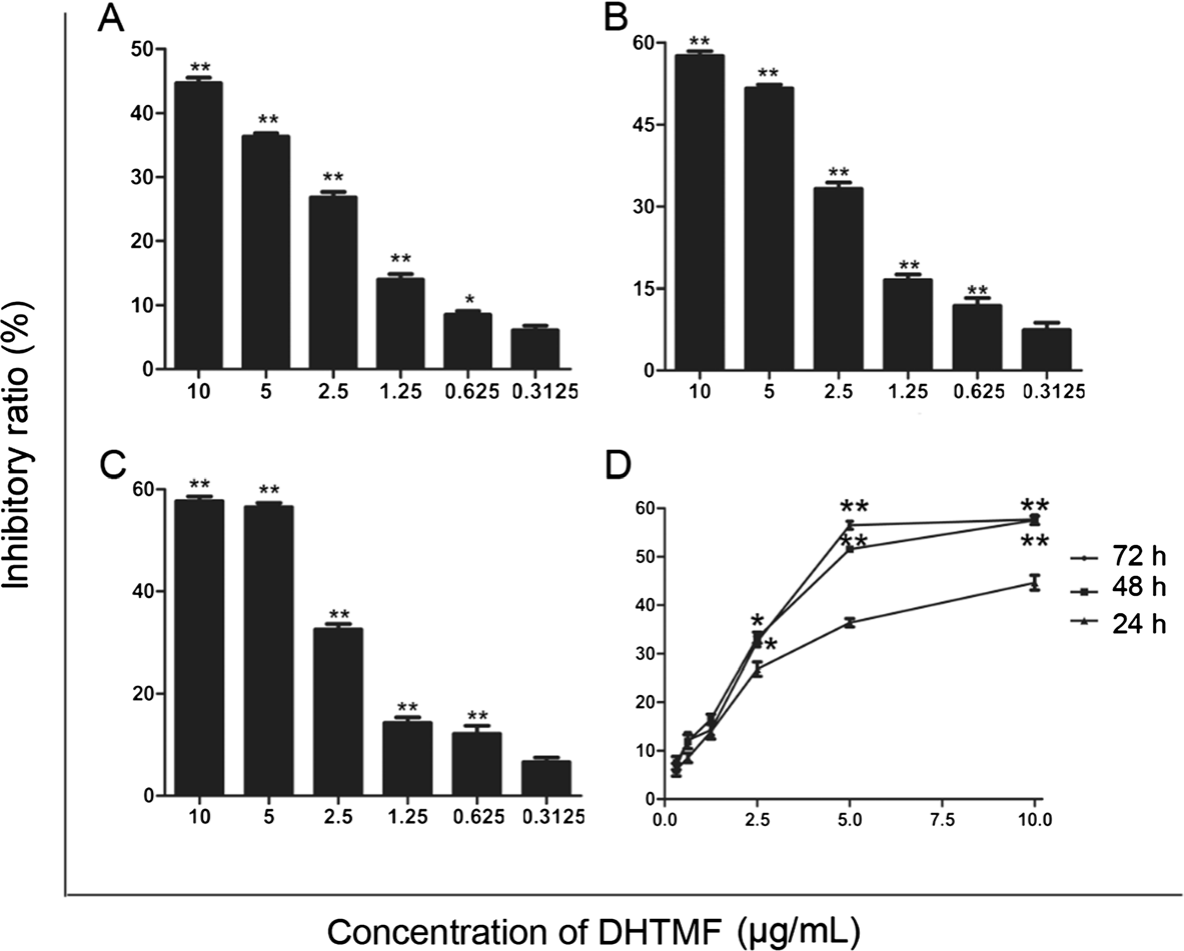
**The inhibitory effect of DHTMF in K562R cells at different treatment times. A**: 24 h, **B**: 48 h, **C**: 72 h. ^**^, *P* < 0.01 vs. 0.3125 μg/mL; **D**: ^*^, *P* < 0.05, ^**^, *P* < 0.01 *vs.* the same concentration for 24 h.

To determine the inhibitory effects of DHTMF, K562R cells were treated with six concentrations of DHTMF for 24, 48, and 72 h, and cell viability was determined by the CCK8 assay. When K562R cells were treated with DHTMF for 24, 48, and 72 h, the inhibitory ratio increased with increasing concentration (*p* < 0.05, *p* < 0.01). DHTMF at concentrations of 24, 48, and 72 h exhibited inhibitory effects with IC_50_ concentrations of 7.85, 5.78, and 5.32 μg/mL, respectively. When comparing with the same concentrations at 24 h, the inhibitory ratio was also significantly increased (*p* < 0.05, *p* < 0.01). As shown in Figure 2, these results suggest that DHTMF suppresses cell viability in a time- and dose-dependent manner.

**Figure 3:**
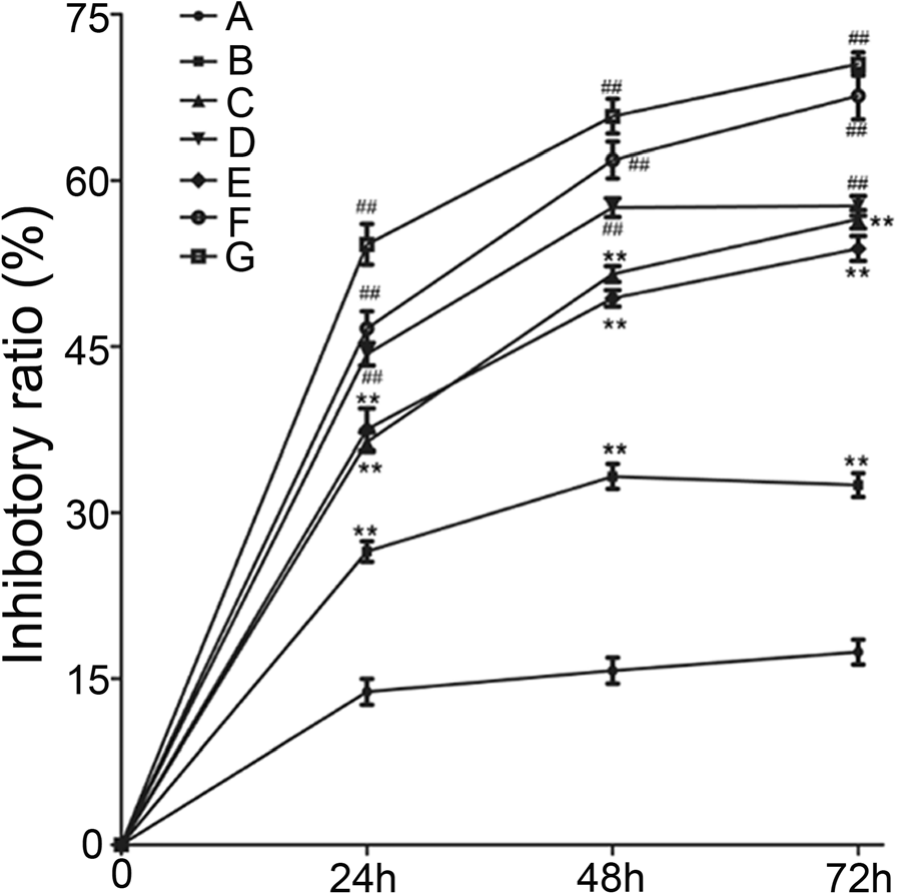
**The inhibitory effects of DHTMF, IM and the combination of IM followed by DHTMF in K562R cells for 24, 48, and 72 h. A**: 1 μmol/L IM; **B**: 2.5 μg/mL DHTMF; **C**: 5 μg/mL DHTMF; **D**: 10 μg/mL DHTMF; **E**: 1 μmol/L IM +2.5 μg/mL DHTMF; **F**: 1 μmol/L IM +5 μg/mL DHTMF; **G**: 1 μmol/L IM +10 μg/mL DHTMF. ^**^, *P* < 0.01 *vs.* 1 μmol/L IM at the same time, ^##^, *P* < 0.01 *vs.* the same concentration as DHTMF alone at the same time.

To further observe the inhibitory effects of DHTMF on K562R cells in the presence or absence of IM, K562R cells were treated with different concentration combinations (1 μmol/L IM, 2.5 μg/mL DHTMF, 5 μg/mL DHTMF, 10 μg/mL DHTMF, 1 μmol/L IM +2.5 μg/mL DHTMF, 1 μmol/L IM +5 μg/mL DHTMF, and 1 μmol/L IM +10 μg/mL DHTMF) for 24, 48, and 72 h, and cell viability was determined by the CCK8 assay. As shown in Figure 3, inhibitory ratio was significantly increased by DHTMF alone and in combination with IM (*P* < 0.01). The inhibitory ratio was greater than 45% when 1 μmol/L IM was combined with 5 μg/mL DHTMF for 24 h. Compared with the same concentration of DHTMF at the same time, the inhibitory activity was also increased in K562R cells (*P* < 0.01). These results suggested that DHTMF combined with IM enhances the inhibitory effects of DHTMF on K562R cells.

### DHTMF alone and in combination with imatinib induces apoptosis in K562R cells

**Figure 4:**
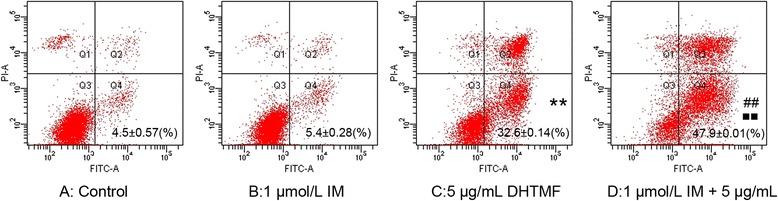
**Induction of apoptosis by DHTMF alone or combination of IM in K562R cells for 24 h treatment. A**: Control; **B**: 1 μmol/L IM; **C**: 5 μg/mL DHTMF; **D**: 1 μmol/L IM+5μg/mL DHTMF. *P* > 0.05, B *vs.* A; ^**^, *P* < 0.01 *vs.* A; ^##^, *P* < 0.01 *vs.* B, ^■■^, *P* < 0.01 *vs.* C.

**Figure 5:**
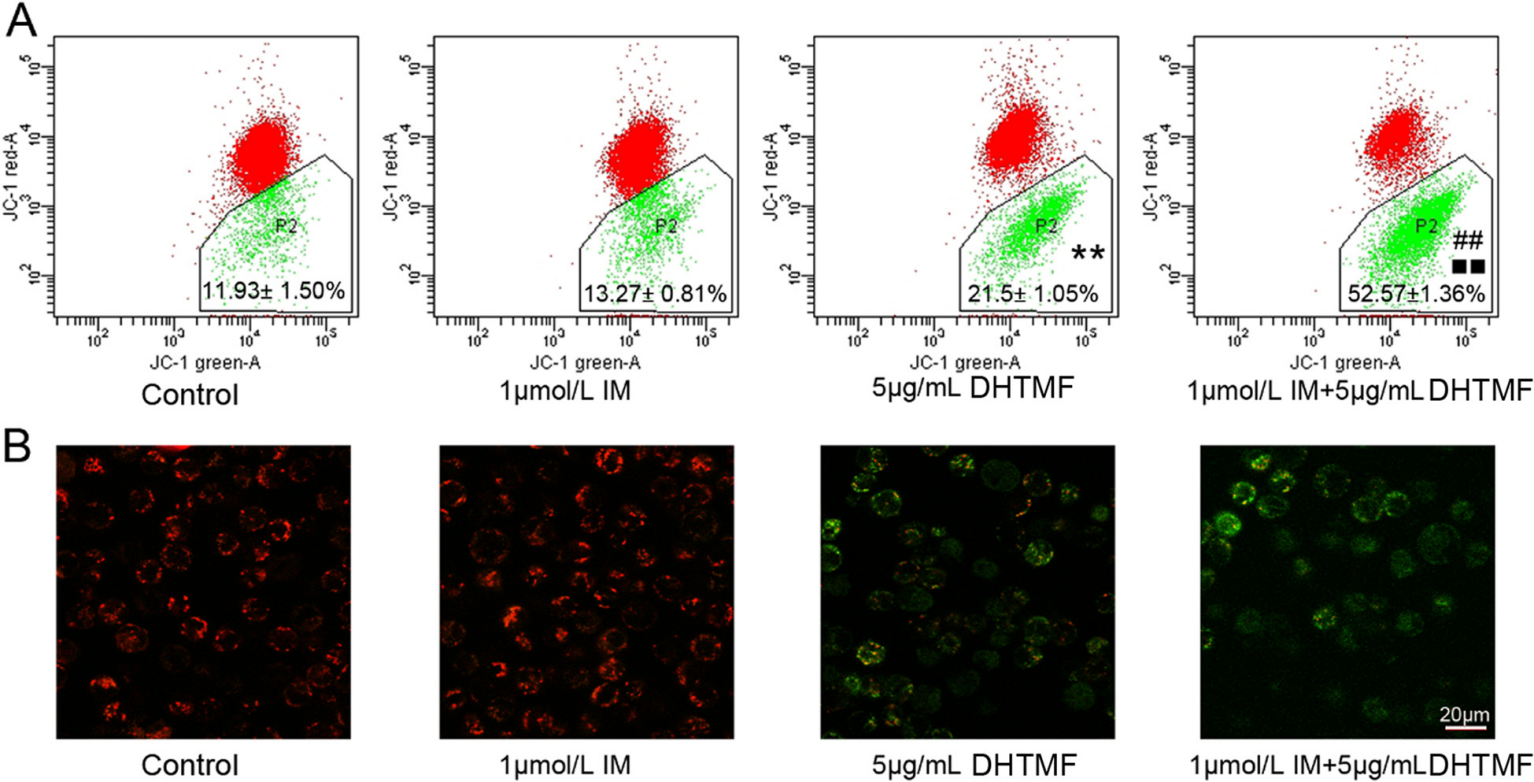
**DHTMF alone or combination of IM induces mitochondria dysfunction in K562R cells. A**: Cells were stained with JC-1 dye and analyzed by flow cytometry.^**^, *P* < 0.01 *vs.* Control, ^##^, *P* < 0.01 *vs.* 1 μmol/L IM, ^■■^, *P* < 0.01 *vs.* 5 μg/mL DHTMF. **B**: DHTMF induces mitochondria dysfunction on K562R cells. Cells were analyzed with a laser confocal microscope, Bar = 20 μm.

To investigate whether the inhibitory effects of DHTMF in K562R cells is associated with apoptosis, treated K562R cells were labeled with AV and PI and analyzed by flow cytometry. Annexin V is an inner membrane protein with a strong affinity for phosphatidylserine. Surface staining of annexin V is used as a general indicator of apoptosis. As shown in Figure 4, most K562R cells were viable in the control group following treatment with different concentration combinations (1 μmol/L IM, 5 μg/mL DHTMF, and 1 μmol/L IM +5 μg/mL DHTMF) for 24 h. However, after incubation with DHTMF alone or in combination with IM for 24 h (Figure 5), the cells displayed an increase in the average apoptosis rate of the cells (Q_4_, AV positive but PI negative) ranging from 4.5 ± 0.57% for the control group and up to 5.4 ± 0.28%, 32.6 ± 0.14%, and 47.9 ± 0.01% for the treatment groups, respectively. K562R cells treated with 5 μg/mL DHTMF or 1 μmol/L IM +5 μg/mL DHTMF had a pronounced increase in apoptosis as indicated by the increase in the AV+/PI − population (4.5% for the control versus 32.6% for 5 μg/mL DHTMF, 5.4% for 1 μmol/L IM vs. 47.9% for 1 μmol/L IM +5 μg/mL DHTMF, and 32.6% for 5 μg/mL DHTMF vs. 47.9% for 1 μmol/L IM +5 μg/mL DHTMF (*P* < 0.01, *P* < 0.01 and *P* < 0.01, respectively).

### DHTMF induces the apoptosis of K562R cells via the mitochondrial apoptotic pathway

To understand the underlying mechanism by which DHTMF alone and in combination with IM induces apoptosis, we investigated changes in the mitochondrial membrane potential and the level of proteins involved in apoptosis.

First, we explored the effects of DHTMF on mitochondrial membrane potential. K562R cells were cultured in different drug concentrations for 24 h and analyzed by flow cytometry and laser confocal microscopy following JC-1 staining (Figure 5A, B). Upon 5 μg/mL DHTMF or 1 μmol/L IM +5 μg/mL DHTMF treatment, the number of cells displaying monomeric green fluorescence increased compared with untreated cells and those exposed to 1 μmol/L IM. These data indicated that the mitochondrial membrane potential is significantly decreased by the 5 μg/mL DHTMF and 1 μmol/L IM +5 μg/mL DHTMF treatments (*P* < 0.01, *P* < 0.01 and *P* < 0.01, respectively). This finding suggests that DHTMF alone and in combination with IM disrupts the mitochondrial membrane potential, resulting in the cytosolic accumulation of monomeric JC-1, which is an indicator of apoptosis via activation of the intrinsic pathway.

**Figure 6:**
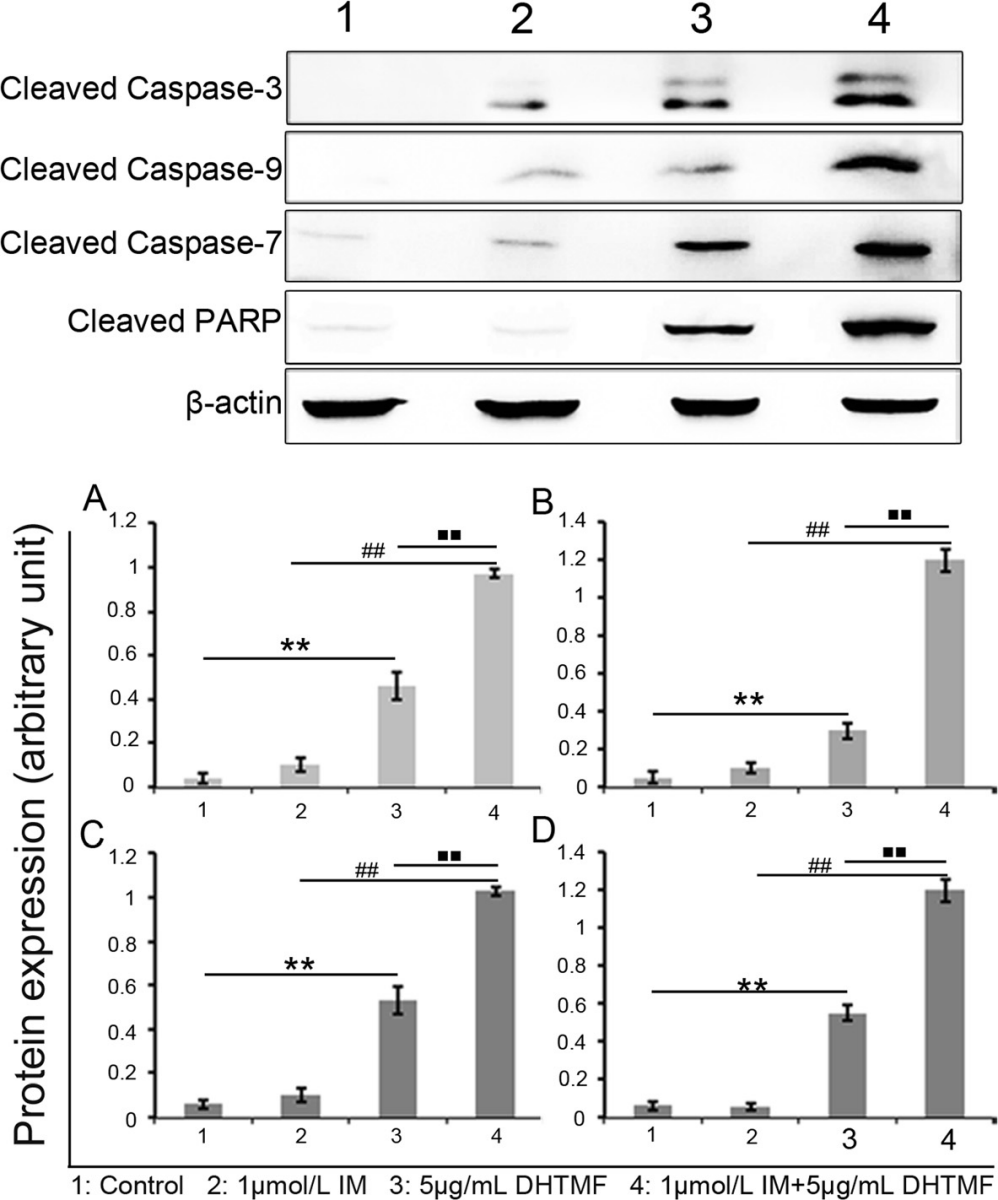
**Western blot analysis in K562R cells.** The relative expression level of the cleaved proteins was analyzed for caspase-3 **(A)**, caspase-9 **(B)**, caspase-7 **(C)** and PARP **(D)**. β-actin was used as a loading control. The results are presented as the ratio of cleaved proteins compared to β-actin intensities. ^**^, *P* < 0.01 *vs.* Control, ^##^, *P* < 0.01 *vs.* 1 μmol/L IM, ^■■^, *P* < 0.01 *vs.* 5 μg/mL DHTMF.

Caspases are crucial players in the induction of apoptotic cell death. Caspase-9 is an initiator caspase that has been implicated in the mitochondria-dependent pathway. To assess the effects of DHTMF alone and in combination with IM on the cleavage of caspases and PARP, cells were treated with different drug concentrations for 24 h, and total cell lysates were prepared for Western blot analysis. As shown in Figure 6, DHTMF affected the increase in the level of cleaved caspase-9, caspase-7, caspase-3, and PARP in K562R cells. In K562R cells treated with 5 μg/mL DHTMF and 1 μmol/L IM +5 μg/mL DHTMF, the immunoreactivity of the cleaved caspases and PARP were dramatically increased (*P* < 0.01, *P* < 0.01 and *P* < 0.01, respectively). These results indicate that DHTMF alone and in combination with IM induces apoptosis in K562R cells via caspase-3, caspase-9, and caspase-7 activation, and DHTMF-induced apoptosis is associated with the mitochondria-dependent pathway.

## Discussion

Treatment with IM is the standard of care for patients newly diagnosed with CML, while several second generation inhibitors, such as dasatinib and nilotinib, have become available with the promise of overcoming some of the mutations associated with acquired resistance in these patients [18]. However, primary TKI therapy resistance and relapse due to the persistence of leukemic stem cells (LSCs) remain a major clinical problem. Moreover, TKI monotherapy is not curative. Recently, combination therapies such as the dual-targeting of the Bcr-Abl and JAK2 activities have been shown to lead to more effective disease eradication, particularly for CML patients at high risk for TKI resistance and disease progression [19,20]. Natural products, such as oridonin, which has been shown to induce the apoptosis of t(8;21) acute myeloid leukemic (AML) cells and inhibit the activity of c-Kit (+) leukemia-initiating cells [21,22], have been considered to play an important role in treating cancer and drug resistance, and they are promising and safe antitumor agents due to their natural origin. DHTMF is a polymethoxyflavone isolated from *Laggera pterodonta* that is a traditional herbal medicine. In a previous study, DHTMF demonstrated antiproliferative activities against a number of tumor cell lines and induced the apoptosis of CNE cells *in vitro* in a time- and dose-dependent manner while exhibiting low cytotoxicity in the two normal cell lines Vero and EVC304 [17]. Therefore, in this study, we used DHTMF in IM-resistant K562 cells (K562R) to investigate its anti-tumor effect. According to this finding, it would be interesting to characterize the effects of DHTMF in anti-leukemia therapy, particularly for the drug resistance of refractory leukemia. In this study, we analyzed the effects of DHTMF in IM-resistant K562 cells (K562R) and found that DHTMF alone or in combination with IM could significantly inhibit proliferation in a time- and dose-dependent manner. As such, the inhibitory ratio was 36.36% when 5 μg/mL DHTMF was used alone for 24 h, while 5 μg/mL DHTMF combined with 1 μg/mL IM could increase the inhibitory activity to 46.63%. These results suggest that DHTMF indeed has inhibitory effects in K562R cells and at the same time improves the sensitivity of K562R cells to IM. By flow cytometry, we confirmed that the percentage of apoptotic cells was significantly increased when K562R cells were treated with 5 μg/mL DHTMF or 1 μmol/L IM +5 μg/mL DHTMF for 24 h. These findings indicate that DHTMF alone or in combination with IM inhibits cell proliferation by inducing apoptosis in K562R cells. There are two main apoptosis pathways: the extrinsic death receptor pathway and the intrinsic mitochondrial pathway [23]. Many polymethoxyflavone induce apoptosis via the mitochondrial apoptosis pathway [24–26]. To further investigate the potential mechanisms of action involved using apoptosis-related analysis, we first observed changes in the mitochondrial membrane potential by flow cytometry and laser confocal microscopy. The results showed that 5 μg/mL DHTMF and 1 μmol/L IM +5 μg/mL DHTMF for 24 h significantly decrease the mitochondrial membrane potential. These results are consistent with the findings of previous studies in which DHTMF was shown to induce the apoptosis of CNE cells in vitro in a time- and dose-dependent manner [17]. These changes suggest that DHTMF is likely to induce apoptosis via the mitochondrial pathway. Caspases are crucial players in the induction of apoptotic cell death. Caspase-9 is an initiator caspase that has been implicated in the mitochondria-dependent pathway [27]. In this study, we demonstrated that DHTMF alone or in combination with IM induces the cleavage of caspase-9, caspase-3, caspase-7, and PARP in DHTMF-treated K562R cells. DHTMF also markedly increased the levels of cleaved caspases and PARP, indicating that the apoptosis mechanism in DHTMF-treated K562R cells might be mediated by activation of the mitochondrial apoptotic pathway and subsequently activate the caspase pathway. Further study is required to elucidate additional mechanisms underlying DHTMF function and determine the exact molecular mechanisms of action of DHTMF in CML cells with IM resistance.

## Conclusions

We demonstrated for the first time that DHTMF alone or in combination with IM inhibits IM-resistant K562R cell proliferation and induces apoptosis via the intrinsic mitochondrial apoptotic pathway. Moreover, DHTMF can improve the sensitivity of K562R to IM. These findings suggest that DHTMF may be a potential therapeutic drug with few side effects in reversing IM-resistance in CML. Whether DHTMF has other effects on Abl-mutated CML cells is worth investigating.

## Methods

### Polymethoxyflavone, cell, and reagents

3,5-Dihydroxy-6,7,3′,4′-tetramethoxyflavone (DHTMF) was isolated from *Laggera pterodonta*, and its structure was identified with spectroscopic methods [28]. IM-sensitive K562 cells (Institutes for Biological Sciences Cell Resource Center, Chinese Academy of Sciences, Shanghai, China) harboring 210 kDa wild-type Bcr-Abl were grown in RPMI 1640 medium (Gibco-BRL, Grand Island, NY, USA) supplemented with 10% fetal calf serum (FCS) (Sijiqing Co., Hangzhou, China) and maintained in a humidified incubator at 37°C and 5% CO_2_. IM-resistant K562R cells (provided by Prof. Jingxuan Pan, Sun Yat-sen University, Guangzhou, China) harboring 210 kDa wild-type Bcr-Abl were routinely maintained in the same medium containing 5 μmol/L IM [29]. Three days before the experiments were performed, IM was no longer added to the media for cell culture.

### Cell proliferation assays

The proliferation of K562R and K562 cells was indirectly assayed using the CCK8 kit (Dojindo, Japan), which stains living cells. Approximately 1 × 10^5^ K562R and K562 cells in 1 mL with 0.1, 1, 10, 100, 1,000 μmol/L IM or different concentrations of DHTMF alone or in combination with IM were incubated in triplicate in 96-well plates. At 24, 48, and 72 h, the CCK8 reagent (10 μL) was added to each well, and the cells were incubated at 37°C for 6 h. The optical density at 450 nm was measured using an automatic microplate reader (Synergy4; Bio-Tek, Winooski, VT, USA). IC_50_ values were determined by plotting compound concentration versus cell viability.

### Apoptosis analysis

At 24 h, K562R cells were treated with or without 1 μmol/L IM, 5 μg/mL DHTMF, or 1 μmol/L IM +5 μg/mL DHTMF. The cells were collected and then prepared with FITC-labeled anti-annexin-V (anti-AV, BD Pharmingen, San Diego, CA, USA) and propidium iodide (PI, Kaiji, Nanjing, China) according to the manufacturer’s protocol and measured by flow cytometry (Beckman Coulter, Fullerton, CA, USA). The data were analyzed using Windows MDI 2.9 software.

### Detection of mitochondrial transmembrane potential using flow cytometry and confocal laser-scanning microscopy

Changes in mitochondrial potential were detected by using 5,5′,6,6′-tetrachloro-1,1′,3,3′ tetraethyl benzimidazolyl carbocyanine iodide/chloride (JC-1), a cationic dye that exhibits potential-dependent accumulation in mitochondria. These changes were indicated by a fluorescence emission shift from red (590 nm) to green (525 nm), and they were analyzed by flow cytometry. At 24 h, K562R cells were treated with or without 1 μmol/L IM, 5 μg/mL DHTMF, and 1 μmol/L IM +5 μg/mL DHTMF. The cells were collected, washed with phosphate-buffered saline (PBS), and analyzed on a flow cytometer. The %Parent green fluorescence cells was calculated for each treatment. At 24 h, K562R cells were treated with or without 1 μmol/L IM, 5 μg/mL DHTMF, and 1 μmol/L IM +5 μg/mL DHTMF. The cells were collected, washed twice with PBS, and stained with JC-1 dye staining. Changes in the mitochondrial transmembrane potential of the cells after JC-1 dye staining were observed with a confocal laser-scanning microscope (LSM 510 META DuoScan; Carl Zeiss, Germany).

### Preparation and analysis of cell lysates by immunoblotting

At 24 h, K562R cells were treated with or without 1 μmol/L IM or 5 μg/mL DHTMF or their combination and were harvested and western blotting was carried out Protein quantification was performed according to conventional methods. Protein samples (30 μg) were boiled with 5 × loading buffer at 100°C for 5 min before being loaded, and they were then electrophoresed in a 12% SDS-PAGE gel at 80 V for 30 min followed by 120 V for 60 min (Bio-Rad). The separated proteins were transferred onto equilibrated PVDF membranes (Invitrogen) using a tank system (Bio-Rad). After blocking with 5% skim milk in TBST for 1 h, the membrane was incubated with individual primary antibodies including β-catenin (mouse anti-actin antibody, 1:1,000 dilution, Lianke, Hangzhou, China), cleaved caspase-3, cleaved caspase-9, cleaved caspase-7 and cleaved PARP (1:1,000 dilution, Cell signaling, Cleaved Caspase Antibody Sampler Kit Cat. No. #9929) at 4°C overnight followed by incubation with goat anti-rabbit or donkey anti-mouse IgG antibodies (Cell signaling, Cleaved Caspase Antibody Sampler Kit Cat. No. #9929; Lianke, Hangzhou, China). Immunoreactive proteins were visualized by chemiluminescence (Lianke, Hangzhou, China), and images were obtained with a Vilber Lourmat system (UVI, UK). The protein expression level was calculated with image quantitative analysis software using β-actin as a reference gene.

### Statistical analysis

Statistical significance was evaluated by one-way ANOVA using SPSS 11.5 statistical software. The results were considered significant at p value less than 0.05.

## Abbreviations

TKIs: Tyrosine kinase inhibitors
DHTMF: 3,5-hydroxy-6,7,3′,4′-tetramethoxyflavone
IM: Imatinib mesylate
CCK8: Counting kit-8
FCM: Flow cytometry
CML: Chronic myeloid leukemia
TK: Tyrosine-kinase
BTK: Bruton's tyrosine kinase
FCS: Fetal calf serum
PI: Propidium iodide
JC-1: 5,5′,6,6′-tetrachloro-1,1′,3,3′ tetraethyl benzimidazolyl carbocyanine iodide/chloride
K562R: IM-resistant K562 cells
